# (3*S*,4*R*)-3-Ethyl-4-hydr­oxy-3-(3-methoxy­phen­yl)-1-methyl­azepanium (2*R*,3*R*)-2,3-bis­(benzo­yloxy)-3-carboxy­propionate

**DOI:** 10.1107/S1600536810013425

**Published:** 2010-04-17

**Authors:** Bo Chao, Xing-Hai Wang, Jian Sun, Zhui-Bai Qiu

**Affiliations:** a Department of Medicinal Chemistry, School of Pharmacy, Fudan University, 826 Zhangheng Road, Shanghai, 201203, People’s Republic of China

## Abstract

The crystal structure of the title compound, C_16_H_26_NO_2_^+^·C_18_H_13_O_8_^−^, is stabilized by an extensive network of classical N—H⋯O and O—H⋯O hydrogen bonding. The crystal structure also shows an ammonium-driven diastereo­isomerism.

## Related literature

For the synthesis of the racemic compound, see: Hao *et al.* (2005 ▶). For conformational studies of seven-membered rings, see: Eliel *et al.* (1994 ▶); Entrena *et al.* (2005 ▶). For a related structure, see: Wang *et al.* (2008 ▶).
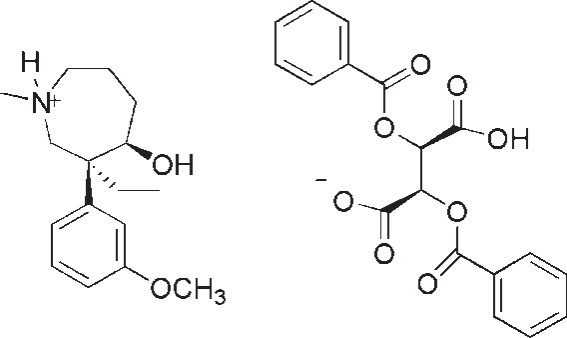


## Experimental

### 

#### Crystal data

C_16_H_26_NO_2_^+^·C_18_H_13_O_8_^−^
*M* *_r_* = 621.66
Triclinic, 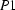

*a* = 7.772 (3) Å
*b* = 14.603 (6) Å
*c* = 15.060 (6) Å
α = 75.313 (6)°
β = 82.182 (6)°
γ = 88.367 (6)°
*V* = 1638.0 (11) Å^3^
*Z* = 2
Mo *K*α radiation
μ = 0.09 mm^−1^
*T* = 295 K
0.25 × 0.15 × 0.10 mm

#### Data collection

Bruker SMART APEX CCD area-detector diffractometer
Absorption correction: multi-scan (*SADABS*; Sheldrick, 1996 ▶) *T*_min_ = 0.977, *T*_max_ = 0.991
8191 measured reflections
5753 independent reflections
2826 reflections with *I* > 2σ(*I*)
*R*_int_ = 0.034

#### Refinement

*R*[*F* ^2^ > 2σ(*F* ^2^)] = 0.048
*wR*(*F* ^2^) = 0.110
*S* = 0.80
5753 reflections
795 parameters
16 restraints
H-atom parameters constrained
Δρ_max_ = 0.19 e Å^−3^
Δρ_min_ = −0.19 e Å^−3^

### 

Data collection: *SMART* (Bruker, 2000 ▶); cell refinement: *SAINT* (Bruker, 2000 ▶); data reduction: *SAINT*; program(s) used to solve structure: *SHELXS97* (Sheldrick, 2008 ▶); program(s) used to refine structure: *SHELXL97* (Sheldrick, 2008 ▶); molecular graphics: *SHELXTL* (Sheldrick, 2008 ▶); software used to prepare material for publication: *SHELXTL*.

## Supplementary Material

Crystal structure: contains datablocks global, I. DOI: 10.1107/S1600536810013425/rk2195sup1.cif

Structure factors: contains datablocks I. DOI: 10.1107/S1600536810013425/rk2195Isup2.hkl

Additional supplementary materials:  crystallographic information; 3D view; checkCIF report

## Figures and Tables

**Table 1:** Hydrogen-bond geometry (Å, °)

*D*—H⋯*A*	*D*—H	H⋯*A*	*D*⋯*A*	*D*—H⋯*A*
N1—H1⋯O16	0.89	1.90	2.719 (6)	151
N2—H2*A*⋯O4	0.89	2.24	2.884 (7)	129
O2—H2*X*⋯O9^i^	0.82	2.18	2.779 (6)	130
O4—H4*X*⋯O18^ii^	0.82	2.12	2.819 (6)	143
O7—H7*X*⋯O9^iii^	0.82	2.53	3.339 (6)	170
O7—H7*X*⋯O10^iii^	0.82	1.91	2.470 (6)	124
O15—H15⋯O17^ii^	0.82	1.62	2.435 (5)	170

Symmetry codes: (i) 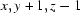; (ii) 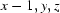; (iii) 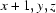.

**Fig. 1.**
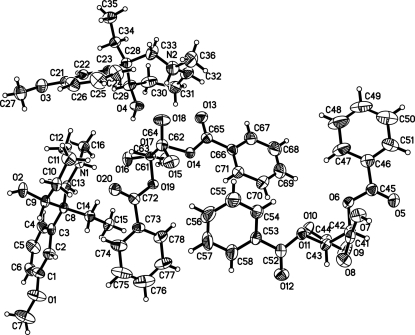
The molecular structure of the title compound with the atom numbering scheme. Displasement ellipsoids are drawn at the 30% probability level. H atoms are presented as a small spheres of arbitrary radius.

## supplementary crystallographic information

### Comment

The overall shape of the title compound can be described as two domains (Fig. 1), *viz*, a multi-substituted azepane and a L-dibenzoyltartrate anion. The absolute configuration of azepane ring atoms were established as C3(*S*) and C4(*R*), according to the reference molecule L-dibenzoyltartaric acid. Molecules are linked by classical N—H···O and O—H···O hydrogen bonds involving all potential donors (Table 1). The 3-methoxyphenyl substituent at C3 is *cis*-configuration to the OH group at C4, resulting in an extended conformation of the cation.

Surprisingly, the solid-state structure of the molecule reveals an ammonium-driven diastereoisomerism. The protonated N1 bears *S*, while the corresponding N2 bears *R*. In addition, the conformations of the two azepane rings are also different, but both of them could be identified as twist-chair forms, which are believed the most preferred conformations in seven-membered rings (Eliel *et al.*, 1994; Entrena *et al.*, 2005). It's worth noting that such phenomenon was not oberserved in its diastereomers, like *D*-tartrate salt of the (3*S*,4*S*)-isomer (Wang *et al.*, 2008). The unique result here could be attributed to the flexibility of the azepane, which can present different conformations in similar energy.

### Experimental

The title compound was prepared by standard procedures upon optical resolution of racemate. The synthesis of the racemic compound was described by Hao *et al.*, (2005). The title compound' configuration is determined, via the known absolute configuration of the anions (2R, 3R), which is a common acid to resolve racemic amines.

### Refinement

The H atoms were positioned geometrically and refined as riding (C—H = 0.93–0.97Å, *N*—H = 0.89Å, O—H = 0.82Å), with *U*_iso_(H) = 1.2*U*_eq_(C) and the three H atoms of the methyl refined as riding (C—H = 0.98Å), with *U*_iso_(H) = 1.5*U*_eq_(C).

Pure diffraction experiment (ratio observed/unique reflections 49%) we explain by weak diffraction of the crystal.

The 1170 Friedel pairs were merged.

### Crystal data

**Table:**

C_16_H_26_NO_2_^+^·C_18_H_13_O_8_^−^	*Z* = 2
*M**_r_* = 621.66	*F*(000) = 660
Triclinic, *P*1	*D*_x_ = 1.260 Mg m^−^^3^
Hall symbol: P 1	Mo *K*α radiation, λ = 0.71073 Å
*a* = 7.772 (3) Å	Cell parameters from 802 reflections
*b* = 14.603 (6) Å	θ = 2.8–19.5°
*c* = 15.060 (6) Å	µ = 0.09 mm^−^^1^
α = 75.313 (6)°	*T* = 295 K
β = 82.182 (6)°	Parallelepiped, colourless
γ = 88.367 (6)°	0.25 × 0.15 × 0.10 mm
*V* = 1638.0 (11) Å^3^	

### Data collection

**Table:**

Bruker SMART APEX CCD area-detector diffractometer	5753 independent reflections
Radiation source: fine-focus sealed tube	2826 reflections with *I* > 2σ(*I*)
graphite	*R*_int_ = 0.034
φ and ω scans	θ_max_ = 25.2°, θ_min_ = 1.4°
Absorption correction: multi-scan (*SADABS*; Sheldrick, 1996)	*h* = −9→9
*T*_min_ = 0.977, *T*_max_ = 0.991	*k* = −16→17
8191 measured reflections	*l* = −18→17

### Refinement

**Table:**

Refinement on *F*^2^	Primary atom site location: structure-invariant direct methods
Least-squares matrix: full	Secondary atom site location: difference Fourier map
*R*[*F*^2^ > 2σ(*F*^2^)] = 0.048	Hydrogen site location: inferred from neighbouring sites
*wR*(*F*^2^) = 0.110	H-atom parameters constrained
*S* = 0.80	*w* = 1/[σ^2^(*F*_o_^2^) + (0.0505*P*)^2^] where *P* = (*F*_o_^2^ + 2*F*_c_^2^)/3
5753 reflections	(Δ/σ)_max_ < 0.001
795 parameters	Δρ_max_ = 0.19 e Å^−^^3^
16 restraints	Δρ_min_ = −0.19 e Å^−^^3^

### Special details

**Table:**

Geometry. All s.u.'s (except the s.u. in the dihedral angle between two l.s. planes) are estimated using the full covariance matrix. The cell s.u.'s are taken into account individually in the estimation of s.u.'s in distances, angles and torsion angles; correlations between s.u.'s in cell parameters are only used when they are defined by crystal symmetry. An approximate (isotropic) treatment of cell s.u.'s is used for estimating s.u.'s involving l.s. planes.
Refinement. Refinement of *F*^2^ against ALL reflections. The weighted *R*-factor wR and goodness of fit *S* are based on *F*^2^, conventional *R*-factors *R* are based on *F*, with *F* set to zero for negative *F*^2^. The threshold expression of *F*^2^ > σ(*F*^2^) is used only for calculating\ *R*-factors(gt) etc. and is not relevant to the choice of reflections for refinement. *R*-factors based on *F*^2^ are statistically about twice as large as those based on *F*, and *R*-factors based on ALL data will be even larger.

### Fractional atomic coordinates and isotropic or equivalent isotropic displacement parameters (Å^2^)

**Table:**

	*x*	*y*	*z*	*U*_iso_*/*U*_eq_	
N1	0.1267 (6)	0.8021 (3)	0.0702 (3)	0.0527 (12)	
H1	0.1645	0.7617	0.1184	0.063*	
O1	0.0083 (7)	1.1593 (4)	0.3418 (4)	0.1107 (19)	
O2	0.2316 (5)	1.0406 (3)	0.0160 (3)	0.0681 (12)	
H2X	0.2882	1.0897	−0.0042	0.102*	
C1	−0.0586 (9)	1.1148 (5)	0.2846 (5)	0.0655 (18)	
C2	0.0504 (8)	1.0513 (4)	0.2549 (4)	0.0613 (17)	
H2	0.1577	1.0400	0.2762	0.074*	
C3	0.0037 (7)	1.0018 (4)	0.1921 (4)	0.0479 (14)	
C4	−0.1594 (9)	1.0219 (4)	0.1656 (4)	0.0673 (18)	
H4	−0.1961	0.9926	0.1233	0.081*	
C5	−0.2679 (9)	1.0837 (5)	0.1996 (6)	0.087 (2)	
H5	−0.3787	1.0925	0.1822	0.104*	
C6	−0.2200 (9)	1.1326 (5)	0.2579 (5)	0.074 (2)	
H6	−0.2938	1.1762	0.2788	0.089*	
C7	−0.0919 (13)	1.2314 (6)	0.3748 (7)	0.141 (4)	
H7A	−0.0979	1.2864	0.3244	0.212*	
H7B	−0.0375	1.2479	0.4222	0.212*	
H7C	−0.2071	1.2079	0.3998	0.212*	
C8	0.1294 (7)	0.9302 (4)	0.1599 (4)	0.0436 (13)	
C9	0.2899 (7)	0.9882 (4)	0.0996 (4)	0.0505 (15)	
H9	0.3217	1.0339	0.1324	0.061*	
C10	0.4512 (7)	0.9319 (4)	0.0800 (4)	0.0586 (16)	
H10A	0.5360	0.9763	0.0396	0.070*	
H10B	0.4975	0.9087	0.1382	0.070*	
C11	0.4455 (8)	0.8496 (5)	0.0383 (5)	0.077 (2)	
H11A	0.4633	0.7925	0.0856	0.092*	
H11B	0.5432	0.8556	−0.0103	0.092*	
C12	0.2830 (8)	0.8353 (5)	−0.0022 (4)	0.0695 (19)	
H12A	0.3069	0.7891	−0.0385	0.083*	
H12B	0.2547	0.8945	−0.0438	0.083*	
C13	0.0350 (7)	0.8796 (4)	0.1046 (4)	0.0497 (15)	
H13A	−0.0719	0.8535	0.1427	0.060*	
H13B	0.0026	0.9266	0.0515	0.060*	
C14	0.1910 (7)	0.8608 (4)	0.2444 (4)	0.0526 (15)	
H14A	0.2729	0.8175	0.2224	0.063*	
H14B	0.2533	0.8966	0.2763	0.063*	
C15	0.0492 (8)	0.8022 (4)	0.3150 (4)	0.0696 (19)	
H15A	−0.0362	0.8438	0.3354	0.104*	
H15B	0.1002	0.7653	0.3671	0.104*	
H15C	−0.0051	0.7608	0.2865	0.104*	
C16	0.0031 (8)	0.7502 (4)	0.0333 (4)	0.0673 (18)	
H16A	−0.0941	0.7285	0.0800	0.101*	
H16B	0.0605	0.6969	0.0163	0.101*	
H16C	−0.0369	0.7916	−0.0201	0.101*	
N2	0.3412 (8)	0.3378 (4)	0.0241 (4)	0.099 (2)	
H2A	0.3398	0.3845	0.0525	0.119*	
O3	0.3518 (6)	0.8128 (3)	−0.2301 (3)	0.0853 (14)	
O4	0.1594 (5)	0.5076 (3)	0.0450 (3)	0.0671 (12)	
H4X	0.0856	0.5362	0.0716	0.101*	
C21	0.4215 (9)	0.7309 (5)	−0.1845 (4)	0.0609 (17)	
C22	0.3197 (8)	0.6523 (4)	−0.1683 (4)	0.0557 (16)	
H22	0.2124	0.6581	−0.1899	0.067*	
C23	0.3720 (7)	0.5635 (4)	−0.1202 (4)	0.0527 (16)	
C24	0.5353 (8)	0.5589 (5)	−0.0888 (5)	0.084 (2)	
H24	0.5776	0.5010	−0.0579	0.101*	
C25	0.6320 (11)	0.6402 (7)	−0.1041 (7)	0.106 (3)	
H25	0.7365	0.6368	−0.0798	0.127*	
C26	0.5800 (10)	0.7256 (6)	−0.1535 (6)	0.084 (2)	
H26	0.6503	0.7790	−0.1659	0.101*	
C27	0.4469 (11)	0.9008 (5)	−0.2537 (5)	0.104 (3)	
H27A	0.4747	0.9145	−0.1983	0.157*	
H27B	0.3771	0.9508	−0.2850	0.157*	
H27C	0.5521	0.8957	−0.2936	0.157*	
C28	0.2545 (7)	0.4771 (4)	−0.1023 (4)	0.0492 (15)	
C29	0.0952 (8)	0.4885 (5)	−0.0333 (4)	0.0651 (18)	
H29	0.0284	0.5432	−0.0624	0.078*	
C30	−0.0287 (8)	0.3998 (6)	0.0010 (5)	0.087 (2)	
H30A	−0.1444	0.4208	0.0197	0.104*	
H30B	−0.0350	0.3719	−0.0504	0.104*	
C31	0.0271 (11)	0.3245 (5)	0.0811 (5)	0.095 (3)	
H31A	0.0270	0.3533	0.1324	0.114*	
H31B	−0.0629	0.2761	0.0996	0.114*	
C32	0.1907 (11)	0.2767 (5)	0.0714 (5)	0.083 (2)	
H32A	0.1785	0.2295	0.0375	0.100*	
H32B	0.2157	0.2435	0.1327	0.100*	
C33	0.3472 (8)	0.3816 (6)	−0.0691 (5)	0.086 (2)	
H33A	0.3012	0.3369	−0.0978	0.103*	
H33B	0.4688	0.3911	−0.0944	0.103*	
C34	0.1910 (8)	0.4722 (4)	−0.1929 (4)	0.0628 (18)	
H34A	0.1113	0.4191	−0.1794	0.075*	
H34B	0.1255	0.5292	−0.2146	0.075*	
C35	0.3281 (9)	0.4621 (5)	−0.2713 (4)	0.088 (2)	
H35A	0.4206	0.5066	−0.2778	0.133*	
H35B	0.2775	0.4745	−0.3278	0.133*	
H35C	0.3733	0.3990	−0.2579	0.133*	
C36	0.5075 (11)	0.2821 (6)	0.0447 (5)	0.122 (3)	
H36A	0.5225	0.2348	0.0103	0.183*	
H36B	0.4987	0.2521	0.1098	0.183*	
H36C	0.6054	0.3245	0.0271	0.183*	
O5	0.9452 (8)	−0.0629 (3)	0.8618 (3)	0.0992 (17)	
O6	0.9088 (5)	0.0795 (3)	0.7676 (2)	0.0524 (10)	
O7	1.2214 (5)	0.1267 (3)	0.7907 (3)	0.0716 (12)	
H7X	1.3074	0.1189	0.8177	0.107*	
O8	1.1174 (5)	0.2261 (3)	0.8756 (3)	0.0726 (13)	
O9	0.5471 (6)	0.1132 (4)	0.9168 (4)	0.117 (2)	
O10	0.5145 (5)	0.1943 (3)	0.7770 (3)	0.0781 (14)	
O11	0.8346 (4)	0.2695 (3)	0.7461 (2)	0.0498 (10)	
O12	0.7405 (5)	0.3877 (3)	0.8101 (3)	0.0749 (13)	
C41	1.0994 (8)	0.1653 (5)	0.8366 (4)	0.0555 (16)	
C42	0.9188 (7)	0.1249 (4)	0.8400 (4)	0.0490 (15)	
H42	0.8902	0.0783	0.8995	0.059*	
C43	0.7844 (6)	0.2025 (4)	0.8329 (4)	0.0456 (14)	
H43	0.7914	0.2338	0.8827	0.055*	
C44	0.5973 (7)	0.1672 (5)	0.8413 (5)	0.0556 (16)	
C45	0.9283 (8)	−0.0146 (5)	0.7871 (5)	0.0623 (17)	
C46	0.9312 (6)	−0.0507 (4)	0.7052 (3)	0.0660 (18)	
C47	0.8973 (7)	0.0068 (3)	0.6206 (4)	0.103 (3)	
H47	0.8763	0.0710	0.6145	0.124*	
C48	0.8949 (9)	−0.0316 (5)	0.5452 (3)	0.153 (5)	
H48	0.8723	0.0069	0.4887	0.184*	
C49	0.9264 (9)	−0.1275 (5)	0.5544 (4)	0.139 (4)	
H49	0.9248	−0.1532	0.5040	0.167*	
C50	0.9603 (7)	−0.1850 (4)	0.6389 (5)	0.130 (4)	
H50	0.9813	−0.2492	0.6451	0.156*	
C51	0.9627 (6)	−0.1466 (4)	0.7143 (4)	0.095 (3)	
H51	0.9853	−0.1851	0.7709	0.114*	
C52	0.8058 (8)	0.3597 (5)	0.7438 (4)	0.0556 (16)	
C53	0.8647 (8)	0.4249 (5)	0.6522 (4)	0.0573 (16)	
C54	0.9568 (11)	0.3910 (5)	0.5829 (5)	0.093 (3)	
H54	0.9810	0.3267	0.5928	0.112*	
C55	1.0134 (16)	0.4525 (7)	0.4984 (6)	0.144 (4)	
H55	1.0783	0.4299	0.4519	0.173*	
C56	0.9737 (16)	0.5470 (8)	0.4832 (6)	0.132 (4)	
H56	1.0101	0.5880	0.4258	0.158*	
C57	0.8808 (14)	0.5815 (6)	0.5517 (7)	0.118 (3)	
H57	0.8537	0.6455	0.5413	0.142*	
C58	0.8279 (10)	0.5192 (5)	0.6369 (5)	0.088 (2)	
H58	0.7665	0.5420	0.6842	0.106*	
O13	0.4031 (7)	0.3360 (3)	0.2308 (3)	0.0842 (14)	
O14	0.4754 (4)	0.4456 (3)	0.3002 (2)	0.0474 (9)	
O15	0.1637 (5)	0.5055 (3)	0.2669 (3)	0.0712 (13)	
H15	0.0680	0.5303	0.2635	0.085*	
O16	0.2626 (5)	0.6433 (3)	0.1736 (3)	0.0685 (12)	
O17	0.8683 (5)	0.5661 (3)	0.2735 (3)	0.0748 (13)	
O18	0.8313 (5)	0.5233 (3)	0.1457 (3)	0.0767 (13)	
O19	0.5475 (4)	0.6277 (3)	0.2977 (2)	0.0465 (9)	
O20	0.6701 (5)	0.7679 (3)	0.2244 (3)	0.0632 (11)	
C61	0.2803 (8)	0.5636 (5)	0.2179 (4)	0.0500 (15)	
C62	0.4637 (6)	0.5201 (4)	0.2181 (3)	0.0412 (13)	
H62	0.4928	0.4951	0.1634	0.049*	
C63	0.5935 (7)	0.5941 (4)	0.2166 (3)	0.0436 (14)	
H63	0.5850	0.6468	0.1622	0.052*	
C64	0.7800 (7)	0.5580 (4)	0.2117 (4)	0.0521 (15)	
C65	0.4332 (7)	0.3582 (5)	0.2999 (4)	0.0540 (16)	
C66	0.4315 (8)	0.2927 (4)	0.3917 (4)	0.0566 (16)	
C67	0.3754 (10)	0.2014 (5)	0.4064 (5)	0.086 (2)	
H67	0.3409	0.1818	0.3573	0.103*	
C68	0.3682 (14)	0.1371 (6)	0.4925 (7)	0.125 (4)	
H68	0.3303	0.0752	0.5013	0.150*	
C69	0.4179 (16)	0.1670 (7)	0.5627 (7)	0.137 (4)	
H69	0.4052	0.1265	0.6218	0.165*	
C70	0.4871 (14)	0.2563 (7)	0.5494 (5)	0.125 (3)	
H70	0.5320	0.2729	0.5974	0.150*	
C71	0.4891 (10)	0.3209 (5)	0.4643 (5)	0.082 (2)	
H71	0.5285	0.3825	0.4557	0.099*	
C72	0.5936 (7)	0.7161 (5)	0.2930 (4)	0.0481 (15)	
C73	0.5347 (6)	0.7444 (3)	0.3800 (3)	0.0556 (16)	
C74	0.5617 (6)	0.8376 (3)	0.3815 (3)	0.079 (2)	
H74	0.6191	0.8797	0.3296	0.095*	
C75	0.5027 (7)	0.8680 (3)	0.4605 (4)	0.117 (3)	
H75	0.5207	0.9304	0.4615	0.141*	
C76	0.4168 (8)	0.8052 (5)	0.5381 (3)	0.131 (4)	
H76	0.3773	0.8255	0.5910	0.157*	
C77	0.3898 (7)	0.7119 (5)	0.5366 (3)	0.124 (3)	
H77	0.3323	0.6698	0.5885	0.148*	
C78	0.4488 (7)	0.6815 (3)	0.4576 (4)	0.089 (2)	
H78	0.4308	0.6191	0.4566	0.106*	

### Atomic displacement parameters (Å^2^)

**Table:**

	*U*^11^	*U*^22^	*U*^33^	*U*^12^	*U*^13^	*U*^23^
N1	0.058 (3)	0.048 (3)	0.051 (3)	0.003 (3)	−0.007 (3)	−0.011 (2)
O1	0.103 (4)	0.123 (5)	0.139 (5)	0.022 (3)	−0.019 (4)	−0.094 (4)
O2	0.056 (3)	0.053 (3)	0.077 (3)	−0.007 (2)	−0.004 (2)	0.015 (2)
C1	0.067 (5)	0.060 (5)	0.076 (5)	0.004 (4)	−0.002 (4)	−0.033 (4)
C2	0.049 (4)	0.069 (5)	0.069 (4)	0.005 (3)	−0.006 (3)	−0.024 (4)
C3	0.039 (4)	0.043 (4)	0.053 (4)	−0.004 (3)	−0.003 (3)	0.004 (3)
C4	0.067 (5)	0.055 (4)	0.086 (5)	0.010 (3)	−0.026 (4)	−0.021 (4)
C5	0.053 (5)	0.087 (6)	0.128 (7)	0.015 (4)	−0.019 (5)	−0.041 (5)
C6	0.060 (5)	0.060 (5)	0.100 (6)	0.007 (4)	0.001 (4)	−0.022 (4)
C7	0.131 (8)	0.134 (9)	0.199 (10)	0.032 (7)	−0.020 (7)	−0.118 (8)
C8	0.040 (3)	0.041 (3)	0.046 (3)	−0.006 (3)	−0.006 (3)	−0.003 (3)
C9	0.046 (4)	0.049 (4)	0.050 (4)	−0.009 (3)	−0.010 (3)	0.003 (3)
C10	0.048 (4)	0.056 (4)	0.063 (4)	0.000 (3)	−0.006 (3)	0.001 (3)
C11	0.039 (4)	0.094 (6)	0.096 (5)	−0.001 (4)	0.007 (4)	−0.028 (4)
C12	0.066 (5)	0.091 (5)	0.047 (4)	0.002 (4)	0.013 (4)	−0.020 (3)
C13	0.049 (4)	0.052 (4)	0.042 (3)	0.005 (3)	−0.003 (3)	−0.003 (3)
C14	0.045 (3)	0.054 (4)	0.054 (4)	−0.003 (3)	0.000 (3)	−0.009 (3)
C15	0.075 (5)	0.069 (5)	0.056 (4)	−0.006 (4)	−0.003 (4)	−0.001 (3)
C16	0.076 (5)	0.059 (4)	0.069 (4)	−0.008 (4)	−0.015 (4)	−0.018 (3)
N2	0.084 (5)	0.095 (5)	0.096 (5)	0.024 (4)	0.031 (4)	−0.005 (4)
O3	0.096 (4)	0.068 (4)	0.083 (3)	−0.018 (3)	0.003 (3)	−0.010 (3)
O4	0.051 (3)	0.085 (3)	0.072 (3)	−0.010 (2)	0.011 (2)	−0.041 (2)
C21	0.071 (5)	0.058 (5)	0.051 (4)	0.002 (4)	0.002 (4)	−0.015 (3)
C22	0.063 (4)	0.052 (4)	0.051 (4)	−0.009 (3)	0.004 (3)	−0.015 (3)
C23	0.046 (4)	0.065 (5)	0.046 (3)	0.002 (3)	0.009 (3)	−0.018 (3)
C24	0.043 (4)	0.088 (6)	0.124 (6)	0.008 (4)	−0.020 (4)	−0.026 (5)
C25	0.068 (5)	0.107 (8)	0.150 (8)	−0.007 (5)	−0.020 (5)	−0.043 (6)
C26	0.065 (6)	0.074 (6)	0.114 (6)	−0.019 (4)	0.005 (5)	−0.033 (5)
C27	0.139 (7)	0.061 (5)	0.104 (6)	−0.045 (5)	0.017 (5)	−0.016 (4)
C28	0.035 (3)	0.058 (4)	0.056 (4)	0.007 (3)	−0.007 (3)	−0.018 (3)
C29	0.050 (4)	0.087 (5)	0.059 (4)	−0.013 (3)	0.004 (3)	−0.024 (4)
C30	0.041 (4)	0.144 (7)	0.082 (5)	−0.015 (4)	0.010 (4)	−0.050 (5)
C31	0.112 (7)	0.075 (5)	0.083 (6)	−0.023 (5)	0.032 (5)	−0.016 (5)
C32	0.101 (6)	0.082 (6)	0.062 (5)	−0.029 (5)	0.013 (4)	−0.018 (4)
C33	0.046 (4)	0.117 (6)	0.069 (5)	0.017 (4)	0.011 (4)	0.011 (4)
C34	0.060 (4)	0.071 (5)	0.056 (4)	−0.001 (3)	−0.001 (3)	−0.016 (3)
C35	0.087 (5)	0.102 (6)	0.075 (5)	0.006 (4)	0.008 (4)	−0.030 (4)
C36	0.103 (7)	0.142 (8)	0.100 (6)	0.075 (6)	−0.008 (5)	−0.002 (5)
O5	0.168 (5)	0.059 (3)	0.070 (3)	0.016 (3)	−0.032 (3)	−0.008 (3)
O6	0.059 (3)	0.053 (3)	0.044 (2)	−0.011 (2)	−0.0099 (19)	−0.007 (2)
O7	0.049 (3)	0.096 (4)	0.075 (3)	−0.005 (3)	−0.007 (2)	−0.031 (3)
O8	0.058 (3)	0.071 (3)	0.097 (3)	−0.006 (2)	−0.017 (2)	−0.032 (3)
O9	0.060 (3)	0.168 (5)	0.082 (3)	−0.051 (3)	−0.005 (3)	0.047 (3)
O10	0.051 (3)	0.116 (4)	0.055 (3)	−0.008 (2)	−0.012 (2)	0.006 (3)
O11	0.050 (2)	0.042 (3)	0.050 (2)	−0.0043 (18)	0.0010 (19)	0.000 (2)
O12	0.062 (3)	0.091 (4)	0.071 (3)	0.012 (2)	0.010 (2)	−0.032 (3)
C41	0.058 (5)	0.050 (4)	0.051 (4)	−0.003 (3)	−0.014 (3)	0.005 (3)
C42	0.044 (4)	0.058 (4)	0.040 (3)	−0.018 (3)	−0.004 (3)	−0.001 (3)
C43	0.034 (3)	0.056 (4)	0.039 (3)	−0.011 (3)	0.003 (3)	−0.001 (3)
C44	0.030 (3)	0.077 (5)	0.047 (4)	−0.012 (3)	0.008 (3)	0.001 (3)
C45	0.066 (5)	0.059 (5)	0.059 (5)	−0.004 (4)	−0.007 (4)	−0.010 (4)
C46	0.059 (4)	0.069 (5)	0.067 (5)	−0.019 (4)	0.004 (4)	−0.015 (4)
C47	0.131 (7)	0.122 (7)	0.063 (5)	−0.046 (6)	−0.002 (5)	−0.035 (5)
C48	0.249 (14)	0.141 (10)	0.078 (6)	−0.078 (9)	0.013 (7)	−0.053 (6)
C49	0.167 (10)	0.178 (11)	0.094 (7)	−0.033 (9)	0.020 (7)	−0.089 (8)
C50	0.099 (7)	0.171 (10)	0.150 (9)	0.005 (6)	−0.010 (7)	−0.102 (9)
C51	0.083 (6)	0.105 (7)	0.115 (7)	0.013 (5)	−0.015 (5)	−0.062 (6)
C52	0.041 (4)	0.055 (5)	0.065 (5)	−0.004 (3)	−0.009 (3)	−0.004 (4)
C53	0.051 (4)	0.058 (5)	0.058 (4)	−0.004 (3)	−0.009 (3)	−0.003 (4)
C54	0.148 (8)	0.058 (5)	0.058 (5)	−0.013 (5)	0.017 (5)	−0.001 (4)
C55	0.268 (14)	0.073 (7)	0.073 (6)	−0.043 (7)	0.029 (7)	−0.004 (5)
C56	0.229 (13)	0.092 (8)	0.067 (6)	−0.058 (7)	−0.018 (7)	0.002 (6)
C57	0.177 (10)	0.051 (5)	0.114 (7)	−0.005 (6)	−0.039 (7)	0.014 (6)
C58	0.101 (6)	0.070 (6)	0.080 (5)	−0.004 (4)	−0.006 (4)	0.005 (4)
O13	0.121 (4)	0.069 (3)	0.063 (3)	−0.019 (3)	−0.004 (3)	−0.020 (3)
O14	0.044 (2)	0.048 (3)	0.050 (2)	0.0049 (19)	−0.0105 (18)	−0.009 (2)
O15	0.039 (3)	0.085 (3)	0.076 (3)	0.012 (2)	−0.001 (2)	0.000 (3)
O16	0.061 (3)	0.055 (3)	0.078 (3)	0.010 (2)	−0.013 (2)	0.004 (2)
O17	0.042 (3)	0.118 (4)	0.072 (3)	0.018 (2)	−0.011 (2)	−0.037 (3)
O18	0.049 (3)	0.110 (4)	0.083 (3)	0.011 (2)	0.001 (2)	−0.052 (3)
O19	0.046 (2)	0.048 (3)	0.045 (2)	0.0038 (19)	0.0048 (18)	−0.0151 (19)
O20	0.063 (3)	0.059 (3)	0.064 (3)	−0.012 (2)	0.004 (2)	−0.014 (2)
C61	0.046 (4)	0.058 (4)	0.046 (4)	−0.005 (3)	−0.005 (3)	−0.013 (3)
C62	0.043 (4)	0.043 (4)	0.038 (3)	0.009 (3)	−0.005 (3)	−0.013 (3)
C63	0.048 (4)	0.044 (4)	0.039 (3)	0.000 (3)	0.005 (3)	−0.015 (3)
C64	0.032 (4)	0.061 (4)	0.058 (4)	0.004 (3)	0.006 (3)	−0.011 (3)
C65	0.045 (4)	0.057 (5)	0.059 (4)	0.001 (3)	0.001 (3)	−0.016 (4)
C66	0.055 (4)	0.048 (4)	0.063 (4)	0.002 (3)	0.005 (3)	−0.012 (3)
C67	0.106 (6)	0.067 (5)	0.081 (5)	−0.015 (4)	0.002 (5)	−0.017 (4)
C68	0.188 (11)	0.065 (6)	0.098 (7)	−0.015 (6)	0.038 (7)	−0.005 (6)
C69	0.228 (12)	0.072 (8)	0.085 (7)	0.009 (7)	0.020 (7)	0.008 (6)
C70	0.220 (11)	0.086 (7)	0.060 (6)	0.013 (7)	−0.010 (6)	−0.007 (5)
C71	0.120 (7)	0.066 (5)	0.061 (5)	0.005 (4)	−0.016 (4)	−0.014 (4)
C72	0.029 (3)	0.054 (4)	0.063 (4)	0.005 (3)	−0.009 (3)	−0.017 (4)
C73	0.049 (4)	0.070 (5)	0.056 (4)	0.008 (3)	−0.014 (3)	−0.027 (4)
C74	0.069 (5)	0.095 (6)	0.092 (5)	0.000 (4)	−0.019 (4)	−0.053 (5)
C75	0.104 (7)	0.140 (9)	0.143 (8)	−0.002 (6)	−0.006 (6)	−0.105 (8)
C76	0.114 (8)	0.201 (12)	0.106 (7)	0.014 (8)	−0.009 (6)	−0.094 (8)
C77	0.163 (9)	0.147 (9)	0.062 (5)	0.013 (7)	0.016 (5)	−0.046 (6)
C78	0.108 (6)	0.097 (6)	0.055 (4)	0.006 (5)	0.006 (4)	−0.017 (5)

### Geometric parameters (Å, °)

**Table:**

N1—C13	1.486 (6)	C35—H35A	0.9600
N1—C16	1.486 (7)	C35—H35B	0.9600
N1—C12	1.519 (7)	C35—H35C	0.9600
N1—H1	0.8900	C36—H36A	0.9600
O1—C1	1.362 (8)	C36—H36B	0.9600
O1—C7	1.441 (8)	C36—H36C	0.9600
O2—C9	1.422 (6)	O5—C45	1.189 (7)
O2—H2X	0.8200	O6—C45	1.339 (7)
C1—C2	1.357 (8)	O6—C42	1.425 (6)
C1—C6	1.367 (9)	O7—C41	1.297 (7)
C2—C3	1.415 (8)	O7—H7X	0.8200
C2—H2	0.9300	O8—C41	1.204 (7)
C3—C4	1.382 (8)	O9—C44	1.228 (6)
C3—C8	1.533 (8)	O10—C44	1.213 (6)
C4—C5	1.364 (9)	O11—C52	1.322 (7)
C4—H4	0.9300	O11—C43	1.433 (6)
C5—C6	1.357 (9)	O12—C52	1.215 (7)
C5—H5	0.9300	C41—C42	1.527 (8)
C6—H6	0.9300	C42—C43	1.513 (7)
C7—H7A	0.9600	C42—H42	0.9800
C7—H7B	0.9600	C43—C44	1.535 (7)
C7—H7C	0.9600	C43—H43	0.9800
C8—C13	1.513 (7)	C45—C46	1.457 (7)
C8—C14	1.537 (7)	C46—C47	1.3900
C8—C9	1.568 (7)	C46—C51	1.3900
C9—C10	1.516 (7)	C47—C48	1.3900
C9—H9	0.9800	C47—H47	0.9300
C10—C11	1.494 (8)	C48—C49	1.3900
C10—H10A	0.9700	C48—H48	0.9300
C10—H10B	0.9700	C49—C50	1.3900
C11—C12	1.517 (8)	C49—H49	0.9300
C11—H11A	0.9700	C50—C51	1.3900
C11—H11B	0.9700	C50—H50	0.9300
C12—H12A	0.9700	C51—H51	0.9300
C12—H12B	0.9700	C52—C53	1.485 (8)
C13—H13A	0.9700	C53—C58	1.365 (8)
C13—H13B	0.9700	C53—C54	1.374 (8)
C14—C15	1.533 (7)	C54—C55	1.382 (10)
C14—H14A	0.9700	C54—H54	0.9300
C14—H14B	0.9700	C55—C56	1.374 (11)
C15—H15A	0.9600	C55—H55	0.9300
C15—H15B	0.9600	C56—C57	1.372 (12)
C15—H15C	0.9600	C56—H56	0.9300
C16—H16A	0.9600	C57—C58	1.389 (10)
C16—H16B	0.9600	C57—H57	0.9300
C16—H16C	0.9600	C58—H58	0.9300
N2—C33	1.381 (8)	O13—C65	1.217 (7)
N2—C32	1.477 (9)	O14—C65	1.328 (7)
N2—C36	1.533 (9)	O14—C62	1.436 (5)
N2—H2A	0.8900	O15—C61	1.277 (7)
O3—C21	1.358 (7)	O15—H15	0.8200
O3—C27	1.440 (7)	O16—C61	1.201 (6)
O4—C29	1.433 (7)	O17—C64	1.260 (7)
O4—H4X	0.8200	O18—C64	1.240 (7)
C21—C22	1.364 (8)	O19—C72	1.332 (6)
C21—C26	1.369 (9)	O19—C63	1.427 (6)
C22—C23	1.394 (7)	O20—C72	1.207 (6)
C22—H22	0.9300	C61—C62	1.544 (7)
C23—C24	1.407 (8)	C62—C63	1.494 (7)
C23—C28	1.525 (8)	C62—H62	0.9800
C24—C25	1.377 (10)	C63—C64	1.527 (7)
C24—H24	0.9300	C63—H63	0.9800
C25—C26	1.362 (10)	C65—C66	1.467 (8)
C25—H25	0.9300	C66—C67	1.370 (8)
C26—H26	0.9300	C66—C71	1.391 (9)
C27—H27A	0.9600	C67—C68	1.389 (10)
C27—H27B	0.9600	C67—H67	0.9300
C27—H27C	0.9600	C68—C69	1.347 (12)
C28—C34	1.532 (7)	C68—H68	0.9300
C28—C29	1.538 (7)	C69—C70	1.381 (12)
C28—C33	1.550 (8)	C69—H69	0.9300
C29—C30	1.570 (9)	C70—C71	1.384 (9)
C29—H29	0.9800	C70—H70	0.9300
C30—C31	1.515 (9)	C71—H71	0.9300
C30—H30A	0.9700	C72—C73	1.482 (7)
C30—H30B	0.9700	C73—C74	1.3900
C31—C32	1.440 (10)	C73—C78	1.3900
C31—H31A	0.9700	C74—C75	1.3900
C31—H31B	0.9700	C74—H74	0.9300
C32—H32A	0.9700	C75—C76	1.3900
C32—H32B	0.9700	C75—H75	0.9300
C33—H33A	0.9700	C76—C77	1.3900
C33—H33B	0.9700	C76—H76	0.9300
C34—C35	1.512 (8)	C77—C78	1.3900
C34—H34A	0.9700	C77—H77	0.9300
C34—H34B	0.9700	C78—H78	0.9300
			
C13—N1—C16	109.8 (5)	N2—C33—H33A	107.1
C13—N1—C12	113.8 (4)	C28—C33—H33A	107.1
C16—N1—C12	110.1 (4)	N2—C33—H33B	107.1
C13—N1—H1	107.6	C28—C33—H33B	107.1
C16—N1—H1	107.6	H33A—C33—H33B	106.8
C12—N1—H1	107.6	C35—C34—C28	116.9 (5)
C1—O1—C7	119.4 (6)	C35—C34—H34A	108.1
C9—O2—H2X	109.5	C28—C34—H34A	108.1
C2—C1—O1	114.1 (6)	C35—C34—H34B	108.1
C2—C1—C6	122.2 (7)	C28—C34—H34B	108.1
O1—C1—C6	123.8 (6)	H34A—C34—H34B	107.3
C1—C2—C3	121.3 (6)	C34—C35—H35A	109.5
C1—C2—H2	119.4	C34—C35—H35B	109.5
C3—C2—H2	119.4	H35A—C35—H35B	109.5
C4—C3—C2	115.2 (6)	C34—C35—H35C	109.5
C4—C3—C8	124.6 (6)	H35A—C35—H35C	109.5
C2—C3—C8	120.1 (5)	H35B—C35—H35C	109.5
C5—C4—C3	121.8 (7)	N2—C36—H36A	109.5
C5—C4—H4	119.1	N2—C36—H36B	109.5
C3—C4—H4	119.1	H36A—C36—H36B	109.5
C6—C5—C4	122.4 (7)	N2—C36—H36C	109.5
C6—C5—H5	118.8	H36A—C36—H36C	109.5
C4—C5—H5	118.8	H36B—C36—H36C	109.5
C5—C6—C1	117.0 (6)	C45—O6—C42	117.6 (4)
C5—C6—H6	121.5	C41—O7—H7X	109.5
C1—C6—H6	121.5	C52—O11—C43	116.6 (4)
O1—C7—H7A	109.5	O8—C41—O7	126.5 (6)
O1—C7—H7B	109.5	O8—C41—C42	119.8 (6)
H7A—C7—H7B	109.5	O7—C41—C42	113.7 (6)
O1—C7—H7C	109.5	O6—C42—C43	108.3 (4)
H7A—C7—H7C	109.5	O6—C42—C41	112.5 (5)
H7B—C7—H7C	109.5	C43—C42—C41	110.5 (5)
C13—C8—C3	107.9 (4)	O6—C42—H42	108.5
C13—C8—C14	111.6 (4)	C43—C42—H42	108.5
C3—C8—C14	109.6 (4)	C41—C42—H42	108.5
C13—C8—C9	112.0 (4)	O11—C43—C42	106.1 (4)
C3—C8—C9	106.8 (4)	O11—C43—C44	111.8 (4)
C14—C8—C9	108.8 (4)	C42—C43—C44	114.0 (5)
O2—C9—C10	111.2 (4)	O11—C43—H43	108.3
O2—C9—C8	106.6 (4)	C42—C43—H43	108.3
C10—C9—C8	116.4 (5)	C44—C43—H43	108.3
O2—C9—H9	107.4	O10—C44—O9	126.9 (6)
C10—C9—H9	107.4	O10—C44—C43	119.7 (5)
C8—C9—H9	107.4	O9—C44—C43	113.4 (5)
C11—C10—C9	121.7 (5)	O5—C45—O6	124.2 (6)
C11—C10—H10A	106.9	O5—C45—C46	123.8 (6)
C9—C10—H10A	106.9	O6—C45—C46	111.9 (6)
C11—C10—H10B	106.9	C47—C46—C51	120.0
C9—C10—H10B	106.9	C47—C46—C45	122.0 (5)
H10A—C10—H10B	106.7	C51—C46—C45	117.9 (5)
C10—C11—C12	118.0 (5)	C48—C47—C46	120.0
C10—C11—H11A	107.8	C48—C47—H47	120.0
C12—C11—H11A	107.8	C46—C47—H47	120.0
C10—C11—H11B	107.8	C47—C48—C49	120.0
C12—C11—H11B	107.8	C47—C48—H48	120.0
H11A—C11—H11B	107.1	C49—C48—H48	120.0
C11—C12—N1	113.6 (5)	C50—C49—C48	120.0
C11—C12—H12A	108.8	C50—C49—H49	120.0
N1—C12—H12A	108.8	C48—C49—H49	120.0
C11—C12—H12B	108.8	C49—C50—C51	120.0
N1—C12—H12B	108.8	C49—C50—H50	120.0
H12A—C12—H12B	107.7	C51—C50—H50	120.0
N1—C13—C8	118.2 (5)	C50—C51—C46	120.0
N1—C13—H13A	107.8	C50—C51—H51	120.0
C8—C13—H13A	107.8	C46—C51—H51	120.0
N1—C13—H13B	107.8	O12—C52—O11	123.9 (6)
C8—C13—H13B	107.8	O12—C52—C53	122.6 (6)
H13A—C13—H13B	107.1	O11—C52—C53	113.5 (6)
C15—C14—C8	116.2 (5)	C58—C53—C54	119.7 (6)
C15—C14—H14A	108.2	C58—C53—C52	119.9 (6)
C8—C14—H14A	108.2	C54—C53—C52	120.4 (6)
C15—C14—H14B	108.2	C53—C54—C55	119.9 (8)
C8—C14—H14B	108.2	C53—C54—H54	120.1
H14A—C14—H14B	107.4	C55—C54—H54	120.1
C14—C15—H15A	109.5	C56—C55—C54	120.0 (9)
C14—C15—H15B	109.5	C56—C55—H55	120.0
H15A—C15—H15B	109.5	C54—C55—H55	120.0
C14—C15—H15C	109.5	C57—C56—C55	120.6 (8)
H15A—C15—H15C	109.5	C57—C56—H56	119.7
H15B—C15—H15C	109.5	C55—C56—H56	119.7
N1—C16—H16A	109.5	C56—C57—C58	118.8 (8)
N1—C16—H16B	109.5	C56—C57—H57	120.6
H16A—C16—H16B	109.5	C58—C57—H57	120.6
N1—C16—H16C	109.5	C53—C58—C57	121.0 (8)
H16A—C16—H16C	109.5	C53—C58—H58	119.5
H16B—C16—H16C	109.5	C57—C58—H58	119.5
C33—N2—C32	118.9 (7)	C65—O14—C62	118.4 (4)
C33—N2—C36	111.5 (5)	C61—O15—H15	109.5
C32—N2—C36	108.3 (6)	C72—O19—C63	117.0 (4)
C33—N2—H2A	105.7	O16—C61—O15	128.4 (6)
C32—N2—H2A	105.7	O16—C61—C62	119.4 (6)
C36—N2—H2A	105.7	O15—C61—C62	112.2 (5)
C21—O3—C27	121.2 (6)	O14—C62—C63	107.0 (4)
C29—O4—H4X	109.5	O14—C62—C61	111.7 (4)
O3—C21—C22	115.3 (7)	C63—C62—C61	109.7 (4)
O3—C21—C26	123.7 (7)	O14—C62—H62	109.5
C22—C21—C26	121.0 (7)	C63—C62—H62	109.5
C21—C22—C23	121.9 (6)	C61—C62—H62	109.5
C21—C22—H22	119.1	O19—C63—C62	107.6 (4)
C23—C22—H22	119.1	O19—C63—C64	110.7 (4)
C22—C23—C24	116.7 (6)	C62—C63—C64	113.0 (4)
C22—C23—C28	120.7 (5)	O19—C63—H63	108.5
C24—C23—C28	122.6 (6)	C62—C63—H63	108.5
C25—C24—C23	119.7 (7)	C64—C63—H63	108.5
C25—C24—H24	120.1	O18—C64—O17	125.7 (5)
C23—C24—H24	120.1	O18—C64—C63	116.1 (6)
C26—C25—C24	122.3 (8)	O17—C64—C63	118.2 (5)
C26—C25—H25	118.9	O13—C65—O14	123.4 (6)
C24—C25—H25	118.9	O13—C65—C66	125.0 (7)
C25—C26—C21	118.3 (7)	O14—C65—C66	111.6 (6)
C25—C26—H26	120.8	C67—C66—C71	118.9 (6)
C21—C26—H26	120.8	C67—C66—C65	119.7 (6)
O3—C27—H27A	109.5	C71—C66—C65	121.4 (6)
O3—C27—H27B	109.5	C66—C67—C68	122.0 (8)
H27A—C27—H27B	109.5	C66—C67—H67	119.0
O3—C27—H27C	109.5	C68—C67—H67	119.0
H27A—C27—H27C	109.5	C69—C68—C67	118.0 (9)
H27B—C27—H27C	109.5	C69—C68—H68	121.0
C23—C28—C34	109.4 (5)	C67—C68—H68	121.0
C23—C28—C29	108.5 (4)	C68—C69—C70	121.8 (9)
C34—C28—C29	108.2 (4)	C68—C69—H69	119.1
C23—C28—C33	113.9 (5)	C70—C69—H69	119.1
C34—C28—C33	105.0 (5)	C69—C70—C71	119.6 (9)
C29—C28—C33	111.7 (5)	C69—C70—H70	120.2
O4—C29—C28	106.9 (5)	C71—C70—H70	120.2
O4—C29—C30	108.9 (5)	C70—C71—C66	119.3 (8)
C28—C29—C30	114.8 (5)	C70—C71—H71	120.4
O4—C29—H29	108.7	C66—C71—H71	120.4
C28—C29—H29	108.7	O20—C72—O19	123.4 (5)
C30—C29—H29	108.7	O20—C72—C73	124.1 (6)
C31—C30—C29	114.2 (6)	O19—C72—C73	112.4 (5)
C31—C30—H30A	108.7	C74—C73—C78	120.0
C29—C30—H30A	108.7	C74—C73—C72	118.1 (4)
C31—C30—H30B	108.7	C78—C73—C72	121.8 (4)
C29—C30—H30B	108.7	C73—C74—C75	120.0
H30A—C30—H30B	107.6	C73—C74—H74	120.0
C32—C31—C30	121.0 (6)	C75—C74—H74	120.0
C32—C31—H31A	107.1	C76—C75—C74	120.0
C30—C31—H31A	107.1	C76—C75—H75	120.0
C32—C31—H31B	107.1	C74—C75—H75	120.0
C30—C31—H31B	107.1	C75—C76—C77	120.0
H31A—C31—H31B	106.8	C75—C76—H76	120.0
C31—C32—N2	115.7 (6)	C77—C76—H76	120.0
C31—C32—H32A	108.3	C78—C77—C76	120.0
N2—C32—H32A	108.3	C78—C77—H77	120.0
C31—C32—H32B	108.3	C76—C77—H77	120.0
N2—C32—H32B	108.3	C77—C78—C73	120.0
H32A—C32—H32B	107.4	C77—C78—H78	120.0
N2—C33—C28	121.1 (6)	C73—C78—H78	120.0
			
C7—O1—C1—C2	177.4 (7)	C52—O11—C43—C42	−144.4 (4)
C7—O1—C1—C6	−1.9 (11)	C52—O11—C43—C44	90.8 (6)
O1—C1—C2—C3	−177.4 (5)	O6—C42—C43—O11	−63.9 (5)
C6—C1—C2—C3	1.9 (10)	C41—C42—C43—O11	59.8 (5)
C1—C2—C3—C4	−1.1 (9)	O6—C42—C43—C44	59.6 (5)
C1—C2—C3—C8	−179.9 (6)	C41—C42—C43—C44	−176.8 (5)
C2—C3—C4—C5	−1.4 (9)	O11—C43—C44—O10	2.5 (8)
C8—C3—C4—C5	177.3 (6)	C42—C43—C44—O10	−117.9 (6)
C3—C4—C5—C6	3.3 (11)	O11—C43—C44—O9	−176.6 (5)
C4—C5—C6—C1	−2.5 (11)	C42—C43—C44—O9	63.1 (7)
C2—C1—C6—C5	−0.1 (10)	C42—O6—C45—O5	3.6 (9)
O1—C1—C6—C5	179.1 (7)	C42—O6—C45—C46	−174.8 (5)
C4—C3—C8—C13	−6.4 (7)	O5—C45—C46—C47	174.8 (6)
C2—C3—C8—C13	172.3 (5)	O6—C45—C46—C47	−6.8 (7)
C4—C3—C8—C14	−128.1 (6)	O5—C45—C46—C51	−2.7 (9)
C2—C3—C8—C14	50.6 (7)	O6—C45—C46—C51	175.7 (4)
C4—C3—C8—C9	114.1 (6)	C51—C46—C47—C48	0.0
C2—C3—C8—C9	−67.2 (6)	C45—C46—C47—C48	−177.5 (5)
C13—C8—C9—O2	47.5 (6)	C46—C47—C48—C49	0.0
C3—C8—C9—O2	−70.3 (5)	C47—C48—C49—C50	0.0
C14—C8—C9—O2	171.4 (4)	C48—C49—C50—C51	0.0
C13—C8—C9—C10	−77.2 (6)	C49—C50—C51—C46	0.0
C3—C8—C9—C10	165.0 (5)	C47—C46—C51—C50	0.0
C14—C8—C9—C10	46.7 (6)	C45—C46—C51—C50	177.6 (5)
O2—C9—C10—C11	−68.5 (7)	C43—O11—C52—O12	0.0 (8)
C8—C9—C10—C11	53.9 (8)	C43—O11—C52—C53	178.8 (4)
C9—C10—C11—C12	12.2 (9)	O12—C52—C53—C58	−7.7 (9)
C10—C11—C12—N1	−71.1 (8)	O11—C52—C53—C58	173.5 (6)
C13—N1—C12—C11	81.3 (6)	O12—C52—C53—C54	172.0 (7)
C16—N1—C12—C11	−154.9 (5)	O11—C52—C53—C54	−6.8 (8)
C16—N1—C13—C8	169.6 (4)	C58—C53—C54—C55	0.8 (12)
C12—N1—C13—C8	−66.4 (6)	C52—C53—C54—C55	−179.0 (8)
C3—C8—C13—N1	−176.4 (4)	C53—C54—C55—C56	−1.7 (15)
C14—C8—C13—N1	−55.9 (6)	C54—C55—C56—C57	1.2 (17)
C9—C8—C13—N1	66.4 (6)	C55—C56—C57—C58	0.1 (16)
C13—C8—C14—C15	−59.7 (6)	C54—C53—C58—C57	0.6 (11)
C3—C8—C14—C15	59.8 (6)	C52—C53—C58—C57	−179.7 (7)
C9—C8—C14—C15	176.2 (5)	C56—C57—C58—C53	−1.0 (14)
C27—O3—C21—C22	−178.5 (5)	C65—O14—C62—C63	−152.1 (4)
C27—O3—C21—C26	3.4 (9)	C65—O14—C62—C61	87.8 (5)
O3—C21—C22—C23	−178.5 (5)	O16—C61—C62—O14	155.2 (5)
C26—C21—C22—C23	−0.3 (9)	O15—C61—C62—O14	−25.7 (7)
C21—C22—C23—C24	0.2 (8)	O16—C61—C62—C63	36.7 (7)
C21—C22—C23—C28	179.4 (5)	O15—C61—C62—C63	−144.1 (5)
C22—C23—C24—C25	1.8 (10)	C72—O19—C63—C62	−152.4 (4)
C28—C23—C24—C25	−177.4 (7)	C72—O19—C63—C64	83.7 (5)
C23—C24—C25—C26	−3.7 (13)	O14—C62—C63—O19	−60.9 (5)
C24—C25—C26—C21	3.6 (12)	C61—C62—C63—O19	60.4 (5)
O3—C21—C26—C25	176.5 (7)	O14—C62—C63—C64	61.7 (5)
C22—C21—C26—C25	−1.5 (10)	C61—C62—C63—C64	−177.0 (5)
C22—C23—C28—C34	49.4 (6)	O19—C63—C64—O18	177.0 (5)
C24—C23—C28—C34	−131.5 (6)	C62—C63—C64—O18	56.2 (7)
C22—C23—C28—C29	−68.5 (6)	O19—C63—C64—O17	−3.3 (7)
C24—C23—C28—C29	110.7 (6)	C62—C63—C64—O17	−124.1 (5)
C22—C23—C28—C33	166.5 (5)	C62—O14—C65—O13	8.2 (8)
C24—C23—C28—C33	−14.4 (8)	C62—O14—C65—C66	−173.1 (4)
C23—C28—C29—O4	−52.6 (6)	O13—C65—C66—C67	−7.2 (9)
C34—C28—C29—O4	−171.2 (5)	O14—C65—C66—C67	174.0 (5)
C33—C28—C29—O4	73.7 (6)	O13—C65—C66—C71	171.5 (6)
C23—C28—C29—C30	−173.5 (6)	O14—C65—C66—C71	−7.2 (8)
C34—C28—C29—C30	67.9 (7)	C71—C66—C67—C68	2.4 (11)
C33—C28—C29—C30	−47.1 (7)	C65—C66—C67—C68	−178.8 (7)
O4—C29—C30—C31	−36.0 (7)	C66—C67—C68—C69	0.3 (14)
C28—C29—C30—C31	83.8 (7)	C67—C68—C69—C70	−5.1 (16)
C29—C30—C31—C32	−61.7 (9)	C68—C69—C70—C71	7.0 (16)
C30—C31—C32—N2	44.2 (10)	C69—C70—C71—C66	−4.1 (13)
C33—N2—C32—C31	−67.6 (9)	C67—C66—C71—C70	−0.4 (10)
C36—N2—C32—C31	163.7 (7)	C65—C66—C71—C70	−179.2 (7)
C32—N2—C33—C28	84.7 (9)	C63—O19—C72—O20	0.4 (7)
C36—N2—C33—C28	−148.2 (7)	C63—O19—C72—C73	178.3 (4)
C23—C28—C33—N2	94.8 (7)	O20—C72—C73—C74	3.3 (7)
C34—C28—C33—N2	−145.6 (7)	O19—C72—C73—C74	−174.6 (3)
C29—C28—C33—N2	−28.6 (9)	O20—C72—C73—C78	−179.2 (4)
C23—C28—C34—C35	61.2 (7)	O19—C72—C73—C78	2.9 (6)
C29—C28—C34—C35	179.3 (5)	C78—C73—C74—C75	0.0
C33—C28—C34—C35	−61.4 (7)	C72—C73—C74—C75	177.6 (4)
C45—O6—C42—C43	−141.2 (5)	C73—C74—C75—C76	0.0
C45—O6—C42—C41	96.4 (6)	C74—C75—C76—C77	0.0
O8—C41—C42—O6	160.0 (5)	C75—C76—C77—C78	0.0
O7—C41—C42—O6	−21.0 (7)	C76—C77—C78—C73	0.0
O8—C41—C42—C43	38.8 (7)	C74—C73—C78—C77	0.0
O7—C41—C42—C43	−142.2 (5)	C72—C73—C78—C77	−177.5 (4)

### Hydrogen-bond geometry (Å, °)

**Table:**

*D*—H···*A*	*D*—H	H···*A*	*D*···*A*	*D*—H···*A*
N1—H1···O16	0.89	1.90	2.719 (6)	151
N2—H2A···O4	0.89	2.24	2.884 (7)	129
O2—H2X···O9^i^	0.82	2.18	2.779 (6)	130
O4—H4X···O18^ii^	0.82	2.12	2.819 (6)	143
O7—H7X···O9^iii^	0.82	2.53	3.339 (6)	170
O7—H7X···O10^iii^	0.82	1.91	2.470 (6)	124
O15—H15···O17^ii^	0.82	1.62	2.435 (5)	170

Symmetry codes: (i) *x*, *y*+1, *z*−1; (ii) *x*−1, *y*, *z*; (iii) *x*+1, *y*, *z*.
